# Supplementary material for: Chromothripsis during telomere crisis is independent of NHEJ, and consistent with a replicative origin
Source: Genome Res. 2019 May;29(5):737–49. doi: 10.1101/gr.240705.118 (PMC6499312; doi:10.1101/gr.240705.118)
Supplement: Supplemental Material [file supp_gr.240705.118_Supplemental_file_1.zip › contigs/annotated_contigs/DB73/contig.2.DB73_length_519_mean_cov_18.0539499037.docx]

**DB73_length_519_mean_cov_18.0539499037**

```
GCAACCGTGGGTGGTGAGAGTCACTCTAGAGGTTTTCCTGAAACTTCTTTCATCTCTGCATTAAAAATAAATAAGTAAACCCAAATGGC
 >chr6:113730647-113731098 - E=2e-252 p=0e+00
ACAGATGTAAAAAAATTAAAAGAATAAAAACTATAAATAAAACCTTAAAATAACATGGTCTTTAATTATTTTACTTTGATTTTAAAGAA

GGACGCTCAATATTCATGGAAAGGAGAGTATATAACTCACATTTAACACATGATACTGTAAAATGGTTATTTCAGAGGCACAAAACCAA

ATCAAACCAAAGAACCACTTTCCTGCTACTTTTACTTATGCATGTGATAGAATGTGAAGAAGGGCAAGGACATTTGTAGGGCAGAGTGA

AAGTAATGGGCCTACCATACCATATTCTCTTTTTTCTTTTCCTTCCTTCCTTCCTT|TTTCTTTCTTTCTTTCTTTCTTTCTTTCTTTC
                                                         >chr19:58938666-58938773 + E=9e
TTTCTT|TCTTTCTCTTTCCTTTCTTTCTTTCTTTCTTTCTTTCTTTCTTTCTTTCTTTCTTTCTTTCTTTCTTTC-53
```
